# Subacute thyroiditis following ginger (Zingiber officinale) consumption

**DOI:** 10.4103/0974-7788.64403

**Published:** 2010

**Authors:** Manjusha Karkare

**Affiliations:** *MD (Ayurveda), Pune, India E-mail: mrkarkare@gmail.com*

Sir,

I read with interest the article on subacute thyroiditis following ginger (*Zingiber officinale*) consumption by Sanavi and Afshar, which was published in the fi rst issue of this year.[1]

Although this article highlights a new aspect of ginger pharmacology, I would like to point out that it is not consistent with Ayurvedic aspects of ginger consumption.

Ayurveda clearly states that ginger is contraindicated in individuals with *pitta prakriti*, in hot seasons and in hot lands like Iran.[2] The dose reported by author (1 g per day) is also too large.[3] The *anupan* (vehicle) of honey is also not the most ideal one for ginger. In fact ginger should have been given with ghee in small quantity, if at all required. The indication for ginger consumption by the patient and her *prakriti* is not given clearly in the report.

Adverse events caused by Ayurvedic drugs have to be considered from the Ayurvedic angle before causality is established. It is necessary to label an adverse reaction due to improper use correctly to avoid misconceptions regarding Ayurvedic drugs and to promote rational use of Ayurvedic medicines.
